# Does higher education shape change in happiness during the COVID-19 pandemic? Evidence from working-age adults in China

**DOI:** 10.3389/fpsyg.2026.1843236

**Published:** 2026-10-05

**Authors:** Huijiang Dai

**Affiliations:** Faulty of Education and Human Development, The Education University of Hong Kong, Hong Kong, China

**Keywords:** COVID-19, DID, happiness, higher education, subjective well-being

## Abstract

The COVID-19 pandemic had profound consequences for subjective well-being. Yet these consequences may have varied by educational attainment. This study aims to investigate whether patterns of happiness change during the COVID-19 pandemic differed between working-age adults with and without higher education. Using nationally representative panel data from the China Family Panel Studies (CFPS) and focusing on the 2018 and 2020 waves (N = 13,203), this research employs a difference-in-differences design to compare changes in self-reported happiness between individuals with and without higher education. By centering higher education as a key stratifying factor, this study extends the higher education literature on how large-scale crises affect happiness. The findings reveal that individuals with higher education experienced a significantly less pronounced decline in self-reported happiness (*β* = 0.186, *p* = 0.003) during the public health crisis, and this apparent higher-education advantage in pandemic-related happiness is highly conditional on socioeconomic and psychosocial circumstances. The results underscore the need for policymakers to address educational disparities when developing mental health and social support strategies for future large-scale crises.

## 1 Introduction

Human well-being has been a long-standing philosophical concern for thousands of years, from ancient reflections on the “good life” to modern debates about quality of life. Over the past decades, behavioral scientists have also turned their attention to this topic, the factors that lead people to subjectively experience their lives as worthwhile and rewarding, otherwise known as “subjective well-being” (SWB) (Diener et al., 2018). Comparative studies across high-income countries (HICs) and low- and middle-income countries (LMICs) indicate that the determinants of SWB may not operate in the same way across contexts, as differences in economic development, income inequality, and welfare-state support may shape how personal and social resources translate into well-being (Haller and Hadler, 2006; Oishi et al., 2022). These contextual differences suggest that the relationship between individual resources and SWB cannot be fully understood independently of the broader economic and institutional environment.

Among the various resources that may shape SWB, education functions both as a personal investment in human capital and as a social institution that shapes life opportunities, making it a key variable in understanding individual well-being (Cockerill, 2014; Jongbloed, 2018). Yet empirical studies of educational impact tend to privilege economic outcomes like employment rate, wages, or other pecuniary returns over human welfare dimensions (Gouthro, 2014; McMahon and Oketch, 2013; Seeberg, 2011). In response, a growing body of recent scholarship has begun to incorporate SWB measures to assess education’s non-economic benefits (Dong et al., 2025; Forgeard et al., 2011; Liao and Li, 2025; Liu et al., 2023; Zepke, 2013). However, differences in how researchers define and measure subjective well-being, whether from only a happiness perspective or life satisfaction indicators, contribute to inconsistent conclusions (Elwick and Cannizzaro, 2017; Michalos, 2017). Clarifying the specific dimension of well-being under investigation is therefore essential for advancing empirical understanding.

The COVID-19 pandemic provides a unique and useful setting for examining whether changes in happiness differed by higher educational attainment. The pandemic generated global uncertainty, job loss, and social isolation (Kraut et al., 2022; Jiang et al., 2020), conditions known to erode both mental health and subjective well-being (Olesen et al., 2013; Virgolino et al., 2022). Although individuals with higher education are often thought to have greater adaptability and economic resources for coping with stress (McMahon, 2009; McMahon and Oketch, 2013), it remains unclear whether these advantages were sufficient to buffer against the unprecedented disruptions of the pandemic. Historical research has shown that graduates entering recessionary job markets often experience long-term declines in income and satisfaction (Oreopoulos et al., 2012), raising the possibility that the pandemic may have disproportionately affected college-educated individuals. Whether similar patterns emerged during the pandemic remains an open question.

These considerations motivate an examination of less understood non-economic effects of education, the necessity to specify the precise well-being dimension, and the limited evidence on whether higher education may support emotional stability during crisis. This study employs a quasi-experimental difference-in-differences (DID) design. The DID approach exploits the pandemic as an exogenous shock to identify the possible impact of higher education experience on individuals’ happiness trajectories (Athey and Imbens, 2017; Wing et al., 2018). Specifically, findings may inform whether happiness trajectories of higher-educated working-age individuals in China differed from those without higher education during the COVID-19 pandemic. The findings of this study may inform policy debates about the non-economic returns to higher education and its potential role in enhancing population resilience to crises.

## 2 Literature review and theoretical framework

### 2.1 Happiness as a component of subjective well-being

Scholars have delineated some basic conceptual frameworks for the definition of well-being or welfare (e.g., Diener et al., 2009; Haybron, 2008; Kesebir and Diener, 2008; Raibley, 2012; Vittersø, 2013). For example, a person enjoys high levels of personal well-being or welfare when their life is going especially well for them (Raibley, 2012). To operationalize well-being for empirical research when facing the diversity of different philosophical perspectives, scholars in psychology have developed the framework of SWB (Diener et al., 2009), which relies on individuals’ self-reported measures.

Within this framework, SWB encompasses two distinct dimensions: (1) life satisfaction, from the cognitive dimension, which reflects cognitive evaluation of one’s life, and (2) happiness, from the affective dimension, which captures affective or emotional experience in daily life (Diener et al., 2009; Diener et al., 2018; Kahneman and Deaton, 2010). While life satisfaction represents a stable and long-term appraisal, happiness is more transient and sensitive to contextual and environmental changes (Diener et al., 2018; Oishi et al., 2016; Stone et al., 2018). In other words, life satisfaction may underscore more profound enjoyment and achievement in life than happiness (Chui and Wong, 2016). Research in psychology and economics further reveals that income and material conditions improve life satisfaction more than emotional happiness, implying that non-pecuniary resources, such as social connection, self-efficacy, and autonomy, play a larger role in sustaining affective well-being (Dolan et al., 2008; Kahneman and Deaton, 2010; Wang and VanderWeele, 2011).

This distinction became particularly salient during the COVID-19 pandemic. Research comparing pre-pandemic and pandemic-era well-being found that while overall life satisfaction showed modest declines, measures of daily emotional experience exhibited much sharper deterioration (Helliwell et al., 2022; Witteveen and Velthorst, 2020). For example, in the United Kingdom, the Office for National Statistics documented a 30.6 percentage-point rise in elevated anxiety and a 12.3 percentage-point fall in happiness ratings between late 2019 and March 2020, while life satisfaction declined more modestly from 7.67 to 6.91 (Vizard et al., 2020).

Importantly, from a measurement standpoint, although single-item measures are sometimes criticized for greater measurement error, they remain the dominant form in large-scale social surveys. Recent evidence from Raudenská (2023) shows that the single-item happiness question exhibits slightly better invariance and broader cross-cultural comparability than the single-item life satisfaction question. This consideration may be particularly relevant in the Chinese context, where some studies (Diener et al., 2018; Hsu et al., 2017; Oishi, 2010) suggest that cognitive evaluations of life satisfaction may be influenced by normative expectations and response styles. Given these considerations, the present study adopts happiness as the central outcome variable. It not only aligns with prior Chinese research on higher education and happiness (Jiang, 2022) but also captures the short-term emotional variations most affected by the pandemic shock.

### 2.2 Education and happiness

Education is often expected to improve life chances and happiness (Cockerill, 2014; Dobosz and Hetmańczyk, 2023; Liang and Sun, 2022; Oreopoulos and Salvanes, 2011), while empirical findings have been mixed. Although many analyses show a positive correlation between educational attainment and subjective well-being (e.g., Blanchflower and Oswald, 2004; Dolan and White, 2007; Salinas-Jiménez et al., 2013; Yang et al., 2022) and education is often assumed to enhance happiness indirectly through income, occupational opportunities, or social status (Araki, 2022; Chen, 2012; Ruiu and Ruiu, 2019), empirical evidence suggests that these pathways are far less robust than educational discourse implies (Helliwell and Putnam, 2004; Jongbloed, 2018). Some studies even find that higher education may increase regret or dissatisfaction when individuals perceive a mismatch between expectations and outcomes, or when overqualification leads to psychological strain (Michalos, 2017; Roese and Summerville, 2005). For instance, highly educated men have been reported to experience higher rates of depression compared with their less educated peers (Chevalier and Feinstein, 2006).

These mixed findings suggest that the relationship between education and well-being may be more context-dependent than previously assumed. Recent research has begun to examine this possibility. Liu et al. (2023), using difference-in-differences analysis, found that China’s higher education expansion policy improved individuals’ subjective well-being during the COVID-19 pandemic, primarily through enhanced social class mobility. However, their study focused on life satisfaction and emphasized structural mechanisms such as social mobility. Similarly, Liao and Li (2025) demonstrate that in the context of credential inflation in China, it is not educational attainment per se but one’s relative educational position that shapes well-being outcomes, further underscoring the conditional nature of education’s effect.

From the standpoint of hedonic adaptation theory, however, it is plausible to assume that education may influence happiness not by permanently elevating the baseline of well-being, but by shaping how individuals systematically adapt to changing circumstances (Diener et al., 2006; Lucas, 2007). In this sense, education may contribute to happiness by equipping individuals with adaptive resources, cognitive flexibility, coping strategies, and social capital that supports emotional stability during a crisis (Almlund et al., 2011; Pekrun, 2018; Wehner and Schils, 2021). This interpretation resonates with several classic educational theories. Human capital theory (Becker, 2002) traditionally views education as an investment that increases productivity and income (Marginson, 2016; Oreopoulos and Petronijevic, 2013; Psacharopoulos and Patrinos, 2018), but its psychological implications extend further: by strengthening problem-solving abilities and perceived control, education can reduce helplessness and foster a sense of efficacy, the key psychological resources for emotional adaptation (Mirowsky, 2017). Moreover, social capital theory (Coleman, 1988; Putnam, 2000) underscores education’s contribution to building trust, networks, and civic participation. Exposure to higher education may expand individuals’ social networks, deepen interpersonal trust, and foster norms of reciprocity and civic engagement. These relational resources constitute a durable social infrastructure that becomes particularly salient under stress. During the pandemic, socially connected individuals could draw on this network for emotional support, practical assistance, and shared sense-making, all of which are known to attenuate the negative emotional consequences of isolation and uncertainty (Helliwell et al., 2023).

Building on these theoretical expectations and a resource-based perspective on happiness under crisis conditions (Kuhn and Brulé, 2019), working-age adults with higher education would be expected to experience relatively smaller declines in happiness during a systemic crisis such as the COVID-19 pandemic when they may enjoy more resources, both psychological and physical. This framework positions education as a potential buffer against acute emotional disruption, a proposition this analysis seeks to examine empirically.

Based on this theoretical reasoning, the study proposes the following hypotheses:

*H1:* Happiness among working-age adults in China changed significantly during the COVID-19 pandemic.

*H2:* Working-age individuals with higher education had higher baseline happiness than those without higher education.

*H3:* Working-age individuals with higher education experienced a smaller decline in happiness between 2018 and 2020 than those without higher education experience.

## 3 Methodology

The present study examines whether the COVID-19 pandemic changed the relationship between higher education and happiness in China. Rather than simply comparing happiness levels between college-educated and non-college-educated adults, the study uses the pandemic as an exogenous shock to ask a more specific question: Did the happiness gap between college-educated and non-college-educated adults widen, narrow, or remain stable during the COVID-19 crisis? To answer this question, this study employs a repeated cross-sectional difference in differences (DID) design using nationally representative CFPS data from 2018 and 2020. The DID is appropriate because it estimates whether the change in happiness over time differs between a treatment group and a control group when the two groups may exist systematical difference in their baseline happiness. In this study, the treatment group consists of adults with college exposure, defined as those who have completed a college degree or enrolled in a college program, while the control group includes adults with no higher education.

By comparing how happiness changed from the pre-pandemic period (2018) to the pandemic period (2020) across these two groups, this framework helps distinguish whether higher education shaped individuals’ psychological response to the COVID-19 shock. A key assumption in DID is that, in the absence of the pandemic, the two groups would have followed similar trajectories over time. To probe this assumption, this research used the pre-pandemic waves (2014 and 2016) data to estimate an event-study model, treating 2018, the last pre-treatment wave, as the reference period. The interaction between survey-year dummies and the education indicator allows the model to capture the differential trajectories of happiness between different educational level groups over time. Statistically insignificant pre-treatment coefficients are generally taken as evidence consistent with the plausibility of the parallel trend assumption. Under this condition, any subsequent divergence in coefficients observed after 2018 can be plausibly attributed to the impact of the COVID-19 pandemic rather than the pre-existing trends.

Formally, the main specification is:

$$\begin{array}{l} Y_{\mathrm{it}}=\alpha +\beta_{1}\;\text{Collegei}+\beta_{2}\;\text{Postt}+\beta_{3}({\text{Post}}_{t}\times {\text{College}}_{i}\;)+\\ \gamma^{T}X_{\mathrm{it}}+\lambda_{u}+\delta_{t}t+\varepsilon_{\mathrm{it}} \end{array}$$

where $Y_{\mathrm{it}}$ is self-reported happiness (0–10); ${\text{College}}_{i}=1$ if the respondent has a college degree; ${\text{Post}}_{t}=1$ for 2020 (0 for 2018); $X_{\mathrm{it}}$ includes gender, age, residence type, marriage situation, region indicators, relationship evaluation, work satisfaction, income, self-rated health, and subjective status; $\lambda_{u}$ denotes unit fixed effects implemented at the region level for repeated cross-sections, and $\delta_{t}$ are year fixed effects. The coefficient β₃, captures the differential change in happiness between college-educated and non-college-educated working-age adults from 2018 to 2020. All models were estimated in R version 4.5.

### 3.1 Data and sample

Data were drawn from the China Family Panel Studies (CFPS) (Institute of Social Science Survey, Peking University, 2022), a nationally representative longitudinal household survey administered biennially since 2010. The CFPS covers over 95% of the Chinese population and provides rich measures of socioeconomic conditions, health, and well-being at both the individual and household levels. Its multistage, stratified, probability sampling design ensures representativeness across 25 provincial-level administrative regions in Mainland China and results in a final dataset comprising 43,975 observations.

The CFPS provides five different types of questionnaires, and this study utilized data from the adult self-report questionnaire, focusing on respondents aged 18 to 65. While the CFPS defines adults as individuals aged 16 and above, this study adopts 18 as the lower bound because, in China, individuals typically complete secondary education and become eligible for the higher education entrance examination at or after the age of 18. Using this threshold ensures that higher educational attainment is meaningfully comparable across respondents. The upper age limit of 65 is chosen to exclude individuals beyond the typical working-age population, for whom retirement, declining labor participation, and non-wage income sources could confound the relationship between education and well-being. After listwise deletion of missing values, the final analytic sample consists of 13,203 college-educated and non-college-educated working-age adults.

### 3.2 Variables

The key independent variable was higher education. Using the CFPS highest credential item, I coded respondents holding a college diploma, associate degree, bachelor degree, or postgraduate degree as exposure to higher education (1), and all lower credentials as no higher education (0). Importantly, respondents who reported having attended a higher education program but not completed a credential were also classified as having higher education. This approach reflects the study’s conceptual focus on exposure to higher education, rather than credential completion. More detailed variable definition information can be seen in Table 1.

**Table 1:** Variable definitions.

Variable	Definition
Happiness	Measured by CFPS item: “Rate your current happiness level.”
Age	Survey year minus the reported year of birth (18–65 years old)
Log income	Natural logarithm of annual personal income
Relationship Evaluation	“Overall, how would you rate your popularity?” as a measure of interpersonal relationship
Health	Measured using the CFPS item on self-rated health
Subjective social status	“How would you rate your local social status” as a measure of subjective status
Resident type	Dummy variable for living area type (according to national statistic department standards)
Work satisfaction	“Overall, how satisfy are you with this job?” as a measure of work satisfaction
Higher Edu	Reported most recent education degree (including current participation)
Marriage	Dummy variable for marriage situation
Gender	Dummy variable for reported sex
Weights	Cross-sectional individual sampling weights provided by CFPS to adjust for unequal selection probabilities and nonresponse, ensuring nationally representative estimates.
Region Central South China	Province includes “Henan,” “Hubei,” “Hunan,” “Guangdong,” “Guangxi,” “Hainan”
Region East China	Province includes “Shanghai,” “Jiangsu,” “Zhejiang,” “Anhui,” “Fujian,” “Jiangxi,” “Shandong”
Region North China	Province includes “Beijing,” “Tianjin,” “Hebei,” “Shanxi,” “Neimenggu”
Region Northeast	Province includes “Liaoning,” “Jilin,” “Heilongjiang”
Region Southwest	Province includes “Chongqing,” “Sichuan,” “Guizhou,” “Yunnan,” “Xizang”
Region Northwest	Province includes “Shaanxi,” “Gansu,” “Qinghai,” “Ningxia,” “Xinjiang”

This table provides the definitions of variables used in the analysis.

The outcome was happiness, measured by the CFPS single-item question on overall happiness (0–10) and treated as a continuous variable with higher values indicating greater happiness. This subjective, single-item overall measure of happiness has demonstrated good reliability and validity in previous empirical research on capturing individuals’ real subjective well-being (Diener et al., 2009; Jiang, 2022; Lu et al., 2020; Tan et al., 2020).

Covariates were selected based on their established associations with happiness and their potential relevance differences in educational experience across demographic, socioeconomic, and geographic groups. To distinguish relatively pre-existing demographic and contextual character from socioeconomic and psychosocial characteristics that may themselves be related to educational experience, covariates were introduced in stages across the regression specification.

The first set comprised demographic and contextual characteristics: gender, age, residence type, marriage status, and region. Age and gender capture demographic heterogeneity (Blanchflower et al., 2023; Mroczek and Kolarz, 1998), while marriage status and urban–rural residence capture important differences in family circumstances and living environments (Liu and Hsieh, 2024; Okulicz-Kozaryn and Valente, 2026). Regional indicators were included to account for persistent geographic differences in economic development, educational opportunities, labor-market conditions, and culture differences across China (Chua et al., 2019).

The second set comprised socioeconomic and psychological characteristics: log-transformed personal income, relationship evaluation, work satisfaction, self-rated health, and subjective social status. These variables capture important dimensions of individuals’ socioeconomic circumstances and psychological feelings that are closely associated with happiness (Bian et al., 2014; Javed et al., 2024; Killingsworth et al., 2023; Li et al., 2023; Marroquín et al., 2017; Satuf et al., 2018; Subramanian et al., 2005). Importantly, these variables were not all treated as unambiguous confounders. Some socioeconomic and psychological characteristics themselves may be associated with, or potentially influenced by, higher education. Therefore, the fully adjusted model estimates the differential association between higher education experience and changes in happiness conditional on the observed demographic, socioeconomic, and psychosocial characteristics.

Table 2 summarizes the characteristics of the analytical sample. The final sample comprised 13,221 observations from the 2018 and 2020 CFPS waves. Mean happiness was 7.43 (SD = 2.04) on the 0–10 scale. Of the respondents, 3,292 had higher education, and 9,929 did not. The descriptive statistics also show variation in demographic and socioeconomic characteristics across the sample. The mean age was 41.19 years (SD = 12.14), while 8,030 respondents lived in urban areas and 5,191 lived in rural areas. Additional characteristics, including interpersonal relationship evaluation, work satisfaction, income, self-rated health, and subjective social status, are reported in Table 2.

**Table 2:** Summary statistics.

Variable	Coding	Observation	Mean	SD	Min	Max
Happiness	0–10, from low to high	13,221	7.43	2.04	0	10
Age	18–65 years old	13,221	41.19	12.14	18	65
Higher Edu	0 = non higher education	9,929				
	1 = higher education (associate degree and above)	3,292				
Gender	1 = male	7,594				
	2 = female	5,627				
Relationship evaluation	0–10, from low to high	13,221	7.06	1.78	0	10
Log income		13,221	10.07	1.65	0	13.82
Health	1–5, from low to high	13,220	2.81	1.10	1	5
Subjective social status	1–5, from low to high	13,221	2.96	1.00	1	5
Work satisfaction	1–5, from low to high	13,221	3.64	0.89	1	5
Resident type	0 = rural	5,191				
	1 = urban	8,030				
Time	0 = 2018	7,313				
	1 = 2020	5,908				
Marriage	0 = unmarried	3,006				
	1 = married (includes cohabit)	10,215				

Observations with missing values on any of the variables entering a given specification were excluded via listwise deletion; SD meant standard deviation.

### 3.3 Analytic strategy

To estimate the difference in happiness from 2018 to 2020 for college-educated versus non-college-educated groups, this study fitted a series of hierarchical weighted least squares models using R 4.5. This approach is particularly appropriate for analyzing large-scale survey data that encompasses diverse population subgroups. The use of sampling weights helps correct for unequal probabilities of selection and potential nonresponse bias, thereby addressing the imbalance in sample representation between college-educated and non-college-educated respondents.

Hierarchical specification facilitates the incorporation of individual-level covariates, allowing the model to account for heterogeneity across demographic, psychological, and socioeconomic strata while preserving the multilevel structure of the survewwy data. Model 1 provides an unadjusted DID estimate. Model 2 adjusts for demographic and contextual characteristics that may account for systematic differences between individuals with and without higher education experience. Model 3 further adjusts for socioeconomic and psychosocial characteristics. Because some variables included in Model 3 may plausibly lie along pathways linking higher education experience to happiness, Model 3 is interpreted as a conditional association rather than a direct effect of higher education.

## 4 Results

### 4.1 Main DID results

Table 3 reports results from three DID specifications (all weighted). In the unadjusted model (Model 1), the treated group exhibited a significantly higher baseline level of happiness (*β* = 0.134, *p* = 0.007). Meanwhile, the higher x post-period interaction was close to zero and not statistically significant (*β* = 0.031, *p* = 0.681). After adding demographic and geographic controls—gender, age, residence type, and marriage situation—in Model 2, the interaction remained insignificant (*β* = −0.045, *p* = 0.545). With additional adjustments for SES and well-being–related covariates—relationship evaluation, work satisfaction, income, health, and subjective social status—Model 3 yields a larger and statistically significant interaction of 0.186 (*p* = 0.003; *N* = 13,203 weighted). This indicates that the higher-education group experienced a 0.186-point smaller decline in happiness relative to the non-higher-education group from 2018 to 2020.

**Table 3:** Difference-in-differences estimates of higher education on happiness (weighted).

Variable	Model 1	Model 2	Model 3
Higher education	0.134** (0.050)	0.214*** (0.053)	−0.131** (0.046)
Post (2020)	0.027 (0.044)	0.048 (0.043)	−0.013 (0.036)
Higher EDUCATION × Post	0.031 (0.075)	−0.045 (0.074)	0.186** (0.063)
*Demographics*			
Gender (ref: Male)	—	−0.078* (0.035)	−0.070* (0.030)
Age	—	−0.015*** (0.002)	−0.022*** (0.002)
Resident (ref: Rural)	—	0.123** (0.039)	0.176*** (0.040)
Marriage (ref: Not married)	—	0.920*** (0.047)	0.864*** (0.040)
*SES and well-being*			
Relationship evaluation	—	—	0.431*** (0.009)
Work satisfaction	—	—	0.342*** (0.017)
Log income	—	—	−0.010 (0.01)
Self-rated health	—	—	0.227*** (0.014)
Subjective social status	—	—	0.304*** (0.016)
Region fixed effects	No	No	Yes
*N*	13,217	13,213	13,203
*R* ^2^	0.001	0.031	0.315

Dependent variable: self-reported happiness (0–10). Standard errors in parentheses. All models use CFPS sampling weights. *p < 0.05; **p < 0.01; ***p < 0.001.

### 4.2 Parallel trends test

Figure 1 and Table 4 below present the pre-pandemic event study used to assess the plausibility of parallel trends. Using 2018 as the reference year, the interaction coefficients for 2014 and 2016 were not statistically significant. For 2014, the estimated coefficient was −0.054 (SE = 0.044; 95% CI [−0.140, 0.032]; *p* = 0.217), while the corresponding estimate for 2016 was −0.292 (SE = 0.224; 95% CI [−0.731, 0.147]; *p* = 0.193). The wide interval in 2016 indicates limited precision, but both intervals span zero and the magnitudes are modest. Overall, there is no evidence of differential pre-trends or anticipatory effects between college and non-college groups prior to 2018.

**Figure 1:**
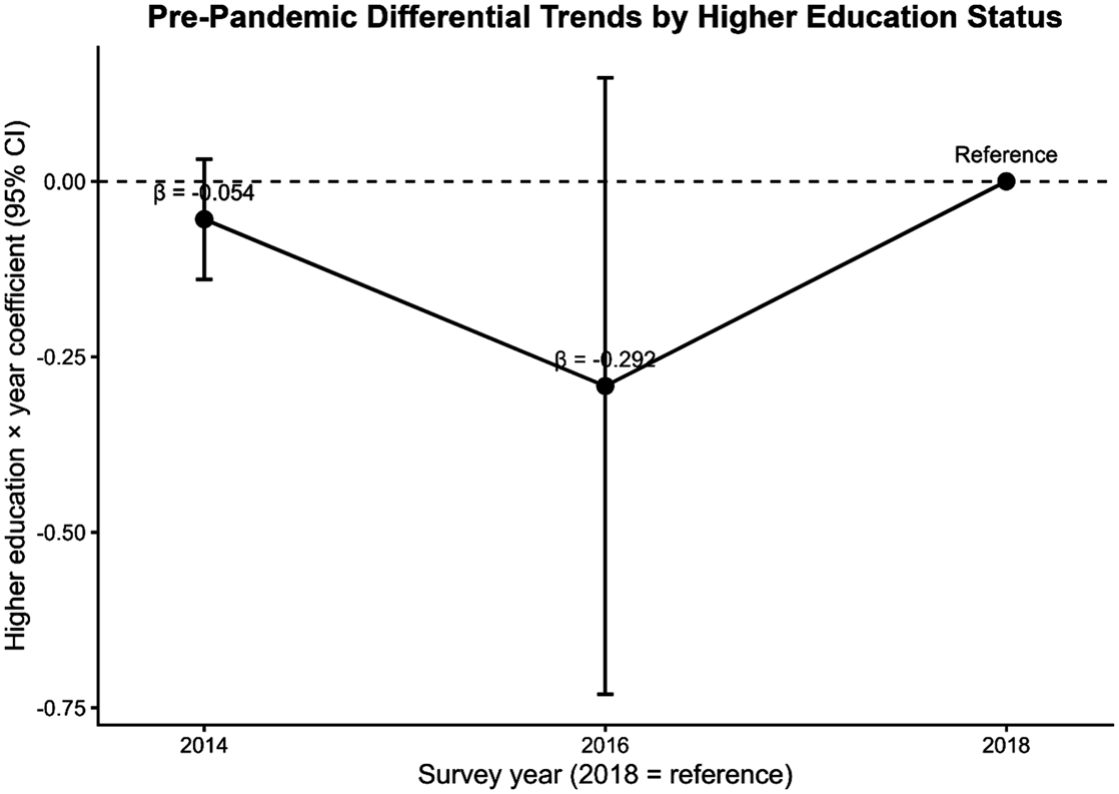
Pre-pandemic differential trends by higher education status. Dots represent estimated coefficients, and lines indicate 95% confidence intervals.

**Table 4:** Parallel trends test (event study).

Year	Estimate	Std. error	95% CI lower	95% CI upper	*p*-value
2014	−0.054	0.044	−0.140	0.032	0.217
2016	−0.292	0.224	−0.731	0.147	0.193

The control variables and weights are consistent with the main specification (Model 3, which includes demographic and SES controls). Due to questionnaire design differences across survey waves, the work satisfaction variable was not consistently available in earlier years and is therefore excluded from the baseline specifications. Marital status was excluded because, after accounting for missingness and sample restrictions, it does not provide sufficient variation to construct comparable groups across time, which would undermine identification in the event-study analysis. The baseline year is 2018. In addition, survey waves prior to 2014 did not include a consistent measure of happiness, rendering them unsuitable for longitudinal analysis.

### 4.3 Weight sensitivity

Table 5 examines whether the DID finding is sensitive to the use of CFPS sampling weights. In the corresponding unweighted specification, the higher education x post pandemic interaction is small and statistically insignificant (*β* = 0.022, SE = 0.07, *p* = 0.749). In contrast, the design-weighted specification produces a larger positive interaction (*β* = 0.186, SE = 0.063, *p* = 0.003).

**Table 5:** Unweighted DID estimates of higher education on happiness.

Variable	*B*	*SE*	*t*	*p*
Higher Education	0.087	0.051	1.688	0.091
Time (2020)	−0.024	0.035	−0.669	0.503
Higher education × Time	0.022	0.070	0.320	0.749
*N*	13,203			
*R* ^2^	0.281			
Adjusted *R*^2^	0.280			

Unweighted OLS estimates with Model 3 covariates. Dependent variable: self-reported happiness (0–10). *p < 0.05; **p < 0.01; ***p < 0.001.

This comparison indicates that the statistical significance of the estimated differential change depends partly on the survey-weighting strategy. Because the CFPS employs a complex sampling design and provides sampling weights to account for unequal selection probabilities and nonresponse, the weighted estimates are retained as the primary population-level estimates. Nevertheless, the difference between weighted and unweighted results indicates that the magnitude and statistical detectability of the estimated association are sensitive to how the survey design is incorporated. The main finding should therefore be interpreted with appropriate caution.

### 4.4 Additional associations

When progressively adding control variables, the coefficient for higher education and happiness became negative and statistically significant (*β* = −0.131, *p* = 0.004). This shift suggests that the observed association between higher education and happiness is substantially attenuated and becomes negative after accounting for socioeconomic and psychosocial characteristics. In other words, higher education may not necessarily correspond directly to greater happiness. This pattern aligns with prior evidence that education can heighten aspirations and stress, offsetting some of its positive effects on emotional well-being (Diener et al., 2018; Michalos, 2017; Roese and Summerville, 2005).

At the same time, among the covariates, interpersonal relationship (*β* = 0.431, *p* < 0.001) and work satisfaction (*β* = 0.342, *p* < 0.001) consistently showed the strongest positive associations with happiness, followed by subjective social status (*β* = 0.304, *p* < 0.001) and self-rated health (*β* = 0.227, *p* < 0.001). In contrast, demographic factors such as gender (*β* = −0.07, *p* = 0.021) and age (*β* = −0.022, *p* < 0.001) exerted relatively limited influence. Urban–rural residence emerges as another meaningful source of heterogeneity. Respondents living in urban areas reported higher levels of happiness than those in rural areas (*β* = 0.176, *p* < 0.001), even before adjusting for other covariates. Meanwhile, regional effects further reveal that residents in northeastern provinces reported higher baseline happiness (*β* = 0.354, *p* < 0.001) than those in the north (*β* = 0.240, *p* < 0.001), eastern (*β* = 0.095, *p* = 0.02), northwestern (*β* = −0.032, *p* = 0.630), and southwest regions (*β* = −0.054, *p* = 0.268). This is noteworthy given that the eastern region is economically more developed, and urban areas generally provide better employment opportunities and living conditions than rural areas.

## 5 Discussion

### 5.1 Summary of findings

Over the past three decades, the Chinese government has pursued an aggressive policy of expanding access to higher education (Jiang, 2022), positioning higher educational credential attainment as both an engine of economic growth and a pathway for upward social mobility. The present study complicates this narrative by showing that the relationship between higher education and happiness is not uniformly positive. Using nationally representative CFPS data, this research finds that the interaction between higher education and the post-pandemic period is positive and statistically significant (*β* = 0.186, *p* = 0.003), whereas the main effect of higher education is negative and significant (*β* = −0.131, *p* = 0.004), and the main effect of the post-pandemic period is negative but not significant (*β* = −0.013, *p* = 0.718). Taken together, these results suggest that higher education did not confer a general happiness advantage in ordinary times, but it was associated with a more favorable happiness trajectory during the COVID-19 period.

At the same time, the negative main effect of higher education indicates that educational attainment may also come with psychological and social costs in the Chinese context. This result contrasts with earlier evidence indicating a modest positive association between education and subjective well-being (Nikolaev, 2018; Tan et al., 2020; Witter et al., 1984). However, it is more consistent with literature emphasizing that the returns to education are shaped by institutional and cultural conditions, including labor market competition, credential inflation, and social comparison processes (Bian et al., 2014; Bourdieu, 2018; Breen and Jonsson, 2005; Buryi and Gilbert, 2014; Card, 1999; Diener et al., 2018; Kim, 2018). In contemporary China, higher education is increasingly massified, but its symbolic and economic value remains tied to intense competition and rising expectations (Zhang and Fan, 2024). As a result, degree holders may face stronger pressure to secure prestigious employment, achieve intergenerational mobility, and justify their educational investment. When these expectations are not fully met, education may generate frustration rather than satisfaction. Overall, one possible interpretation of the negative coefficient for higher education points to the dual character of higher education as both a resource and a source of strain in stratified and highly competitive environments.

The non-significant main effect of the post-pandemic period is also noteworthy, because it diverges from studies reporting substantial declines in well-being during COVID-19 (Helliwell et al., 2022; Witteveen and Velthorst, 2020). Several explanations are plausible. First, evidence from Google Trends data shows that pandemic-related declines in well-being indicators exhibited partial mean reversion within weeks to months of initial lockdowns (Brodeur et al., 2021), a pattern consistent with hedonic adaptation theory (Diener et al., 2006; Lucas, 2007). By the time the 2020 CFPS data were collected, acute emotional disruption may have already partially subsided for much of the sample. Second, the CFPS 2020 data were collected throughout the year, meaning respondents surveyed later in 2020 may have captured a period of relative stabilization rather than the peak of disruption. Consequently, the absence of a uniform happiness decline at the population level does not contradict the central finding of this study; rather, it shifts the analytical focus toward differential trajectories of happiness across higher- and non-higher-education groups.

The interaction effect is therefore the central substantive finding. It suggests that higher education moderates the relationship between the pandemic period and happiness, even though education was not associated with higher baseline happiness overall. This result helps reconcile conflicting information from the literature about the relationship between higher education and happiness. The findings indicate that in relatively stable contexts, the burdens attached to higher education may offset some of its benefits. During periods of external shock, however, the adaptive capacities associated with higher education appear to become more salient, producing a relative advantage in maintaining happiness.

The association observed for the control variables and comediators provide additional context for interpreting the main findings. Urban residency, strong interpersonal relationships, higher self-rated health, job satisfaction, and perceived social status all exhibited strong positive associations with happiness (Lei et al., 2015; Clark et al., 2019). These findings suggest that higher education may operate indirectly by expanding access to urban opportunities, strengthening social networks, and increasing health awareness and occupational stability. Conversely, gender and income were not significant predictors in the fully adjusted model (Cai et al., 2023; Easterlin et al., 2021), highlighting the relative universality of higher education’s protective or buffering benefits across demographic groups. Regional heterogeneity was also observed, with residents in East, North, and Northeast China reporting higher happiness compared to other regions, suggesting that geographic infrastructure and policy contexts may modulate the buffering function of education (see Table 6).

**Table 6:** Regional fixed effects on happiness (Model 3).

Region	Coefficient	*SE*	*t*	*p*
Central South China	—	—	—	—
East China	0.095*	(0.041)	2.321	0.0203
North China	0.240***	(0.045)	5.284	<0.001
Northeast	0.354***	(0.050)	7.052	<0.001
Northwest	−0.032	(0.067)	−0.481	0.630
Southwest	−0.054	(0.049)	−1.108	0.268

Estimates from Model 3 (full specification). Dependent variable: self-reported happiness (1–10). Central South China serves as the reference category. *p < 0.05; **p < 0.01; ***p < 0.001.

### 5.2 Limitations

Several limitations should be noted. First, the analysis focused only on short-term effects of the pandemic shock. Second, although the difference-in-differences design addresses observed confounding, unmeasured heterogeneity remains a concern; the present analysis treats higher education as a homogeneous category. Substantial heterogeneity exists across academic fields, institutional tiers, and student populations, which may yield divergent buffering effects (Gerber and Cheung, 2008). Third, happiness is measured using a single-item rating that captures primarily the affective dimension of subjective well-being rather than its cognitive-evaluative aspects, such as life satisfaction. Consequently, the analysis cannot disentangle which dimensions of subjective well-being are most influenced by higher education. Meanwhile, measurement limitations, including omitted variables, may therefore affect the robustness of the estimated relationships, and several covariates are conceptually and empirically related, which may make it difficult to interpret their coefficients as fully independent associations. Fifth, generalizability is bounded by China’s distinctive institutional context, including its post-1999 enrollment expansion (Jiang, 2022; Liu et al., 2023) and uniquely stringent COVID-19 response, limiting extrapolation to settings where both higher education structures and pandemic management differ markedly. Finally, the 2020 data are treated as a uniform post-period despite considerable temporal variation in lockdown intensity and economic disruption across months and regions, potentially masking heterogeneous shock and adaptation dynamics.

### 5.3 Implications of study findings

For practice, these findings underscore the need for higher education institutions and policymakers to view student subjective well-being not as a peripheral concern. Institutions should invest in durable mental-health infrastructures, expand access to trained professionals, and embed resilience, emotional regulation, and help-seeking skills into curricula and co-curricular programming. Because many institutions lack long-term intervention monitoring and often rely heavily on non-health professionals (Hou et al., 2024; Winzer et al., 2018), institutions should also develop stronger systems for follow-up care and outcome evaluation. Such an institutional approach would position higher education not merely as a provider of mental-health services, but as an environment that can foster students’ social, educational, and psychological resources for adapting to periods of uncertainty.

From a policy perspective, the findings indicate that the returns to higher education should be evaluated not only in terms of employment and earnings but also in terms of happiness. Policymakers should incorporate higher education related happiness indicators into higher education evaluation systems, provide dedicated funding for campus mental health services, and align the higher education system with broader social protection measures such as financial aid, health support, and employment assistance. In the post-pandemic era, policies that strengthen health promotion, social support infrastructure, and social recognition may help amplify the well-being benefits of education.

### 5.4 Future research directions

Future research can extend this study in several ways. Collecting and analyzing individual-level longitudinal data rather than relying on cross-sectional evidence may better capture changing processes and reduce bias caused by unobserved individual differences. Moreover, future studies could investigate the heterogeneity across academic fields, institutional tiers, and student subgroups, as these factors may yield divergent effects on happiness. In addition, the generalizability of the present findings is limited by the unique institutional context and pandemic response. This underscores the value of cross-national and cross-cultural comparative studies. For example, researchers can compare the relationship between higher education and happiness in countries with different higher education systems and welfare regimes, such as China, the United States, and Nordic countries. Furthermore, based on the extra finding that higher education does not always lead to a higher level of happiness, future research may rely on qualitative research to explain the complex relationship between higher education and happiness.

## 6 Conclusion

Accordingly, if higher education serves a non-economic protective role, then expanding equitable access becomes a critical strategy for strengthening population-level buffering capacity. Nevertheless, because these benefits are conditional, the Chinese government must recognize that policies should also confront the structural inequalities that determine who can access higher education and how effectively educational attainment can be translated into well-being gains (Liu and Ma, 2018; Jinzhong, 2010; Sheng, 2014). Addressing this challenge requires targeted interventions to broaden educational opportunities in rural and underdeveloped regions, increased investment in targeted scholarships, and job-market reforms to ensure that higher education functions not only as a pathway to economic mobility but also as a buffer against psychological stress (State Council of the People’s Republic of China, 2024; Ma et al., 2022).

## Data Availability

Publicly available datasets were analyzed in this study. This data can be found here: doi: 10.18170/DVN/45LCSO.
